# Immunogenicity and safety of CoronaVac vaccine in children and adolescents (Immunita-002, Brazil): A phase IV six-month follow up

**DOI:** 10.1038/s41598-025-94596-9

**Published:** 2025-07-02

**Authors:** Camila Amormino Corsini, Priscila Fernanda da Silva Martins, Priscilla Soares Filgueiras, Adelina Júnia Lourenço, Ana Esther de Souza Lima, Sarah Vieira Contin Gomes, Wander de Jesus Jeremias, Pedro Augusto Alves, Gabriel da Rocha Fernandes, Luciana Lisboa Mota e Castro, Andrea Teixeira-Carvalho, Ana Carolina Campi Azevedo, Caroline De Almeida Leitao Curimbaba, Daniela Aparecida Lorencini, Eolo Morandi Junior, Victor Mattos da Silva, Maria Célia Cervi, Marcos de Carvalho Borges, Maurício Lacerda Nogueira, Guilherme Rodrigues Fernandes Campos, Paulo Roberto Lopes Correa, Taciana Malheiros Lima Carvalho, Jordana Grazziela Alves Coelho dos Reis, Erik Vinícius de Sousa Reis, Leda dos Reis Castilho, Poliana Remundini de Lima, João Paulo Resende do Nascimento, Jaquelline Germano de Oliveira, Olindo Assis Martins Filho, Immunita Team, Lívia Paulucci Cavalcanti de Andrade, Isabela Camargos, Érica Louback de Oliveira, Ana Clara Almeida, Daniel Alvim Pena de Miranda, Raquel Amorim, Camila Bedran Pedretii, Thaís Bárbara de Souza Silva, Dayane Andriotti Otta, Viviane Cristina Fernandes dos Santos, Ágata L. Ribeiro, Luís Adan F. Andrade, Thaís de Fátima S. Moraes, Tulio M. Lima, Daniel P. B. de Abreu, Renata G. F. Alvim, Rafaella Fortini Queiroz e Grenfell

**Affiliations:** 1 Oswaldo Cruz Foundation (FIOCRUZ), 1715 Augusto de Lima Avenue, Belo Horizonte, Minas Gerais 30190-002 Brazil; 2 Department of Pharmacy, Federal University of Ouro Preto (UFOP), 27, Nove, Bauxita, Ouro Preto, 35400-000 Brazil; 3 Instituto Butantan, São Paulo, São Paulo 05503-900 Brazil; 4 Faculty of Medicine, University of São Paulo (USP), 455 Doutor Arnaldo Avenue, São Paulo, São Paulo 01246-903 Brazil; 5 Serrana Clinical Research Center. 438, 13 de Maio, Centro, Serrana, São Paulo 14150-000 Brazil; 6 Faculty of Medicine of São José Do Rio Preto (FAMERP), 5416 Brigadeiro Faria Lima Avenue, São José Do Rio Preto, São Paulo 15090-000 Brazil; 7 Hospital de Base, 5544 Brigadeiro Faria Lima Avenue, São José Do Rio Preto, São Paulo State 15090-000 Brazil; 8 Department of Pathology, University of Texas Medical Branch, 301 University Blvd, Galveston, TX 77555 USA; 9 Belo Horizonte Municipal Health Department (SMS), 2336 Afonso Pena Avenue, Belo Horizonte, 30130-012 Brazil; 10 Federal University of Minas Gerais (UFMG), 6627 Presidente Antônio Carlos Avenue, Belo Horizonte, Minas Gerais 31270-901 Brazil; 11 Cell Culture Engineering Laboratory (COPPE), Federal University of Rio de Janeiro (UFRJ), 550 Pedro Calmon Avenue, Rio de Janeiro, Rio de Janeiro 21941-598 Brazil; 12 Department of Infectious Diseases, College of Veterinary Medicine, University of Georgia (UGA), 501 DW Brooks Drive, Athens, GA 30602-7387 USA

**Keywords:** Vaccine, CoronaVac, SARS-CoV-2, COVID-19, Neutralizing antibodies, Antibodies kinetics, Cellular markers, Infectious diseases, Viral infection, Vaccines, Inactivated vaccines

## Abstract

Vaccines are essential for the prevention and control of several diseases, and monitoring the immune response generated by vaccines is crucial. The immune response generated by vaccination against SARS-CoV-2 in children and adolescents is not well defined in terms of the intensity and medium to long-term duration of protective immunity, which may indicate the need for booster doses and could support decisions in public health. The study aims to evaluate the immunogenicity and safety of an inactivated SARS-CoV-2 vaccine (CoronaVac) in a two-dose primary protocol in children and adolescents aged 3 to 17 years old in Brazil. Participants were invited to the research at two public healthcare centers located in Serrana (São Paulo) and Belo Horizonte (Minas Gerais), Brazil. They underwent medical interviews to gather their medical history, including COVID-19 history and medical records. Physical exams were conducted, which included measurements of weight, blood pressure, temperature, and pulse rate. Blood samples were obtained from the participants before vaccination, 1 month after the first dose, and at 1, 3, and 6 months after the second dose. These samples were followed up using a virtual platform to monitor post-vaccination reactions and symptoms of COVID-19. The SARS-CoV-2 genome from swab samples of COVID-19 positive individuals was sequenced using NGS. Total antibodies were measured by ELISA, and neutralizing antibodies to the B.1 lineage and Omicron variant (BA.1) were quantified by PRNT and VNT assays. The cellular immune response was evaluated by flow cytometry through the quantification of systemic soluble immune mediators. The follow-up of 640 participants showed that CoronaVac was able to significantly induce the production of total IgG antibodies to SARS-CoV-2 and the production of neutralizing antibodies to the B.1 lineage and Omicron variant. Additionally, a robust cellular immune response was observed, characterized by a wide release of pro-inflammatory and regulatory mediators in the early post-immunization moments. Adverse events recorded so far have been mild and transient, except for seven serious adverse events reported on VigiMed. The results indicate a robust and sustained immune response induced by CoronaVac in children and adolescents for up to six months, providing evidence to support the safety and immunogenicity of this effective immunizer.

## Introduction

The development of safe and effective vaccines for SARS-CoV-2 was crucial for managing the COVID-19 pandemic and will continue to be the primary tool for limiting the virus’s spread^1^.

The CoronaVac, an inactivated SARS-CoV-2 vaccine utilizing the inactivation technology developed by Sinovac Biotech and produced by the Butantan Institute in Brazil, has played a significant role in the global effort to combat the COVID-19 pandemic^2^. In phase I and II studies, CoronaVac induced seroconversion in 97% of adults and 96.9–100% of the elderly^3,4^. Phase III studies demonstrated that CoronaVac’s efficacy in preventing symptomatic cases, hospitalizations, and COVID-19-related deaths ranged from 50.7–83.5%, 83.7–87.5%, and 86.3–100%, respectively^5,6^. In a randomized study using a stepped-wedge model, CoronaVac’s efficacy in preventing the same outcomes was 80.5%, 95%, and 94.9%, respectively^7^.

Initially authorized for emergency use by various health authorities, including Brazil’s National Health Surveillance Agency (Anvisa), in January 2021, where it was first administered to elderly individuals and healthcare professionals. The initial regimen involved two doses administered with an interval of 2 to 4 weeks between them. By January 2022, approximately 85 million doses (600 SU in 0.5 ml) of this vaccine had been administered to the Brazilian population^8^. In the same year, Anvisa expanded this vaccination protocol to include children and adolescents nationwide^9,10^. Despite CoronaVac no longer being the primary vaccine in Brazil, accumulated data from its use continue to inform public health strategies worldwide^11^.

The immune response generated by COVID-19 vaccines is still under investigation, and further studies are necessary to gain a better understanding of protection against SARS-CoV-2 following vaccination^11^, especially in children and adolescents. In this age group, the immune response triggered by vaccination is not clearly defined in terms of its intensity and duration of protection in the medium and long term, as well as its ability to neutralize distinct variants of concern (VOCs). This information may indicate the need for booster shots and require healthcare managers to make informed decisions^12^.

Based on this, the aim of the present study was to comprehensively assess both humoral and cellular responses and evaluate the effectiveness of the two-dose primary protocol of CoronaVac vaccination in children and adolescents in Brazil. The data generated throughout this phase 4 monitoring study in children and adolescents will contribute significantly to enhancing our understanding of the protective response, effectiveness, and safety of COVID-19 vaccines in this specific age group.

## Methods

### Ethics statement and participants

This study received approval from the Research Ethics Committee involving Human Subjects at the Oswaldo Cruz Foundation, the Ethics Committee of Hospital das Clínicas of the Faculty of Medicine of Ribeirão Preto, University of São Paulo, and the National Council of Ethics in Research (CAAE 55,183,322.6.0000.5091), and it was supervised by Anvisa. Inclusion criteria comprised children and adolescents aged 3 to 17 years who were unvaccinated for COVID-19 and who, with agreement from their parents or guardians, willingly participated in the study and signed the informed consent and assent forms (ICF/IAF). Regarding the exclusion criteria, in accordance with Anvisa’s approval, the agency did not impose any restrictions on the administration of CoronaVac for immunosuppressed children aged 3 to 5 years. However, children and adolescents aged 6 to 17 years with immunosuppression were not included in this study. Informed consent was obtained from the parent(s) and/or legal guardian(s) of all participants in this study. As for deferral criteria, participants with suspected SARS-CoV-2 infection waited up to 72 h for confirmation of the diagnosis. Upon confirmation, vaccination was postponed for a minimum of four weeks. All methods described in this study were performed in accordance with relevant guidelines and regulations, according to Resolution No. 251 of August 7, 1997, from the National Health Council of Brazil^13^.

### Participant recruitment, sample collection, and follow-up

Participants were invited to join the research at two public healthcare centers located in Serrana (São Paulo) and Belo Horizonte (Minas Gerais), Brazil. After obtaining informed consent and applying inclusion and deferral criteria, each participant, accompanied by their parents or guardians, underwent a medical interview to collect comprehensive medical history, any prior symptomatic SARS-CoV-2 infection, and details about concurrent medications, whether related or unrelated to their medical history. Subsequently, physical examinations were conducted, including measurements of weight and vital signs such as systolic and diastolic blood pressure, axillary temperature, and pulse rate. For the definition of obesity, the WHO’s criteria for overweight/obesity were used, as recommended by the Brazilian Ministry of Health^14^. Following these examinations, participants underwent peripheral blood sampling and were guided through the vaccination procedure at the healthcare facility.

A total of 640 participants who met the inclusion criteria were followed for six months after completing the two-dose primary protocol of CoronaVac, administered with a 28-day interval between doses.

Peripheral blood samples were collected were collected from March 2022 to July 2023 at multiple time points: prior to vaccination, on the day of the second dose administration, and at one month, three months, and six months post-second dose, relative to the date of administering the second dose, along with the previously described physical examinations. A 10 ml whole blood sample was obtained via venous puncture from each participant according to biosafety standards and subsequently centrifuged at 3.000 g/5 min to obtain serum for immunogenicity analyses.

**Fig. 1:**
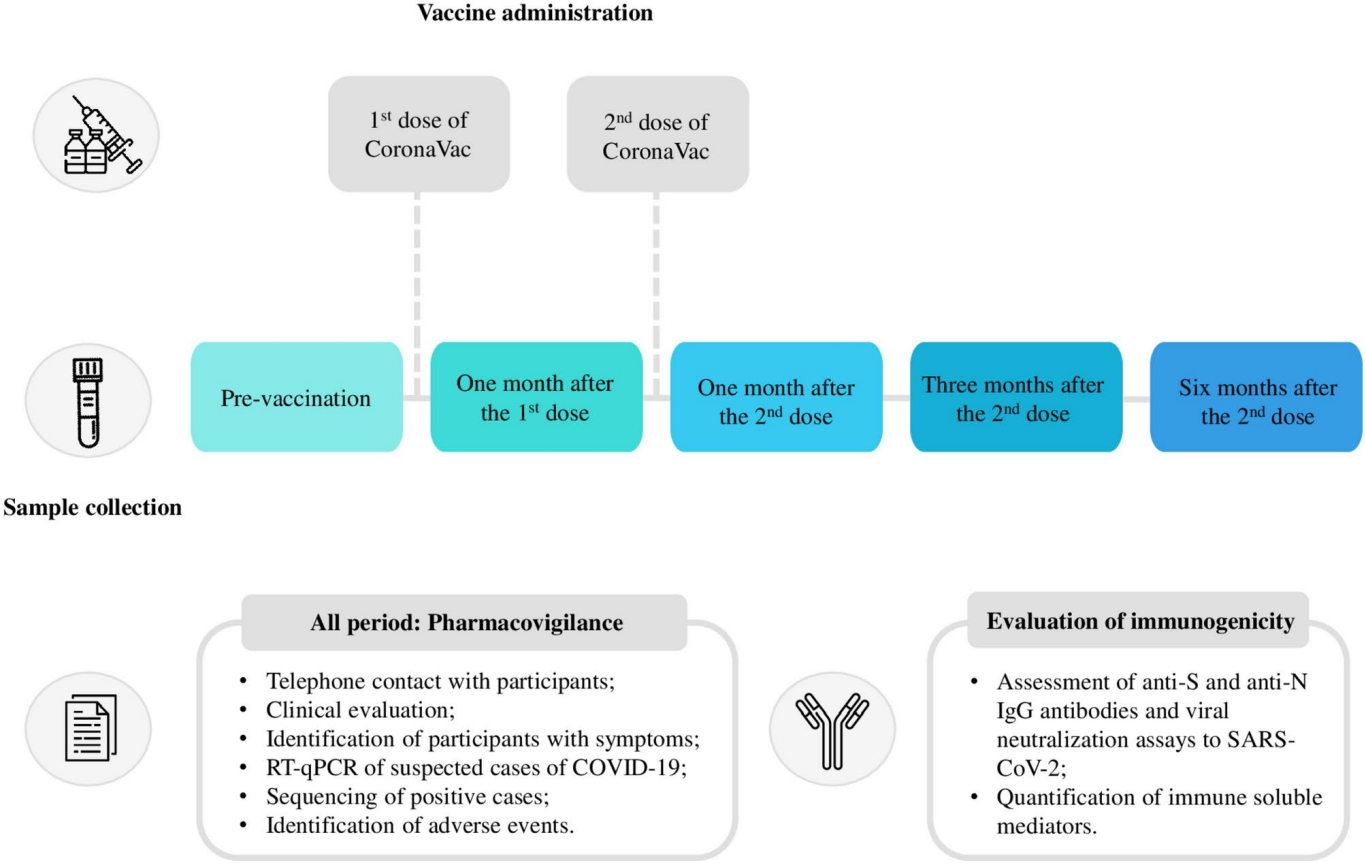
Schematic representation of the Immunita-002 study design.

All participants were monitored daily via a virtual platform for seven days to report any post-vaccination adverse events and continuously for reporting any suspected COVID-19 symptoms. Participants showing symptoms suggestive of the disease underwent medical evaluation, including nasopharyngeal swab collection for confirmation of diagnosis via RT-qPCR. Positive samples underwent next-generation sequencing (NGS) for further analysis. Data on participants’ hospitalizations or adverse events were obtained from medical reports and/or participants’ medical records. The study design is illustrated in schematic representation in Fig. 1.

### Assessment of anti-S and anti-N IgG antibodies and viral neutralization assays to SARS-CoV-2

All serum samples obtained from individuals participating in the study underwent testing for specific total IgG antibodies to the Spike (S) and Nucleocapsid (N) proteins of SARS-CoV-2 at all study sample collection times. These proteins, used as antigens, were generated in stable recombinant HEK293 cells^15^. Antibody detection was performed using standardized ELISA assays^1^, which had been validated by the National Institute of Health Quality Control of Oswaldo Cruz Foundation (INCQS/Fiocruz).

For the assessment of SARS-CoV-2 neutralizing antibodies, a subset of participants who had not previously been diagnosed with COVID-19 was evaluated at time points before vaccination, and at one, three, and six months after the second dose of CoronaVac. Two distinct assays were employed for this purpose: the plaque reduction neutralization test (PRNT) assessed neutralizing antibodies against SARS-CoV-2 (B.1), while the viral microneutralization assay (VNT50) evaluated neutralizing antibodies against the Omicron variant (BA.1)^1,9^.

### Quantification of immune soluble mediators

The Luminex Bio-Plex Pro™ human cytokines platform (Bio-Rad, #M500KCAF0Y)^16^ was utilized to quantify systemic soluble biomarkers, enabling the investigation of 27 analytes, including chemokines (CXCL8, CCL11, CCL3, CCL4, CCL2, CCL5, and CXCL10), pro-inflammatory cytokines (IL-1β, IL-6, TNF-α, IL-12, IFN-γ, IL-15, and IL-17), regulatory cytokines (IL-1Ra, IL-4, IL-5, IL-9, IL-10, and IL-13), and growth factors (FGF-basic, PDGF, VEGF, G-CSF, GM-CSF, IL-7, and IL-2). Measurements were conducted using a Bio-Plex 200 instrument (Bio-Rad).

### Identification of adverse events and serious adverse events following vaccine administration

All participants underwent seven-day monitoring following each dose of the COVID-19 vaccine, facilitated through telephone communication, to identify any adverse events (AEs) and serious adverse events (SAEs).

In this study, AEs included any unfavorable medical incidents reported by vaccinated participants, which may not necessarily have had a direct causal connection with vaccine administration. In contrast, SAEs were defined as adverse events resulting in death, risk of death at the time of the event, hospitalization, or extension of existing hospitalization, significant or persistent disability significantly disrupting routine functions, or clinically significant events arising from necessary medication use during a medical intervention aimed at preventing death, significant disability, or hospitalization.

During in-person follow-up visits and within the initial seven days after each vaccine dose, participants were queried about specific signs and symptoms (solicited AEs) and encouraged to report any additional signs and symptoms (unsolicited AEs). The intensity of solicited AEs was categorized on a numerical scale ranging from 1 to 4, detailed in Supplementary table 1 (local events) and Supplementary table 2 (systemic events), developed according to the Toxicity Grading Scale for Healthy Adult and Adolescent Volunteers Enrolled in Preventive Vaccine Clinical Trials by the United States Food and Drug Administration^17^ and the Common Terminology Criteria for Adverse Events—Version 5.0 by the United States National Cancer Institute^18^. Similarly, unsolicited AEs were graded based on this numerical scale, as per Supplementary table 3, in accordance with the same guidelines. The highest reported intensity for an AE, until resolution or outcome, was used for study analyses.

Furthermore, AEs were classified based on their causal relationship with the CoronaVac, following adapted classification from the Uppsala Monitoring Centre of the World Health Organization (UMC, WHO), as outlined in Supplementary table 4. All local reactions post-vaccination were considered AEs definitively linked to the vaccine. All solicited and unsolicited AEs identified during the initial two weeks after each vaccine dose administration were documented, regardless of their causal association with the vaccine. These events were meticulously recorded in a spreadsheet using the RedCap software, with immediate notification of SAEs to the VigiMed platform of the Brazilian National Health Surveillance Agency^19^.

### COVID-19 confirmation and viral sequencing

In case of suspected COVID-19 during the study period, purified RNA from nasopharyngeal swab samples was used for RT-qPCR following the Charité/Berlin and Centers for Disease Control and Prevention (CDC, USA) protocols^20^. SARS-CoV-2 positive samples with a cycle threshold (Ct) value < 34 underwent NGS sequencing^1,21^. The sequences obtained were promptly deposited in the Global Initiative on Sharing All Influenza Data (GISAID)^22^.

### Statistical analysis and data mining

Data analyses were conducted using GraphPad Prism® software. The results obtained from antibody titer quantification underwent statistical analysis using the Kruskal–Wallis test and Mann–Whitney normality tests, with a significance level set at p < 0.05. Soluble mediator signatures were derived by transforming serum levels, initially expressed as continuous variables (pg/mL). For clinical monitoring records, the platforms Cytoscape and RedCap were employed.

## Results

### Demographic data of the patients

A total of 640 participants aged 3 to 17 years (median age 9 years) were consented and enrolled in the study. Of the participants, 325 (50.75%) were male. The most common comorbidities were asthma in 42 (6.56%) participants, obesity in 18 (2.81%), and bronchitis in 8 (1.25%), as detailed in Table 1.

**Table 1:** Demographic data of participants in the study.

	N	%
Age, years		
3–11	562	87.8
12–17	78	12.2
Gender		
Male	325	50.8
Female	315	49.2
Locality, home city		
Serrana, São Paulo, Brazil	547	85.5
Belo Horizonte, Minas Gerais, Brazil	93	14.5
COVID-19 prior to vaccination		
Yes	88	13.75
No	552	86.25
Main reported comorbidities		
Asthma	42	6.6
Obesity	18	2.8
Bronchitis	8	1.25

### Immunogenicity response: Kinetics of total anti-S and anti-N IgG antibodies, neutralizing antibodies to live SARS-COV-2, and immune soluble mediators

**Fig. 2:**
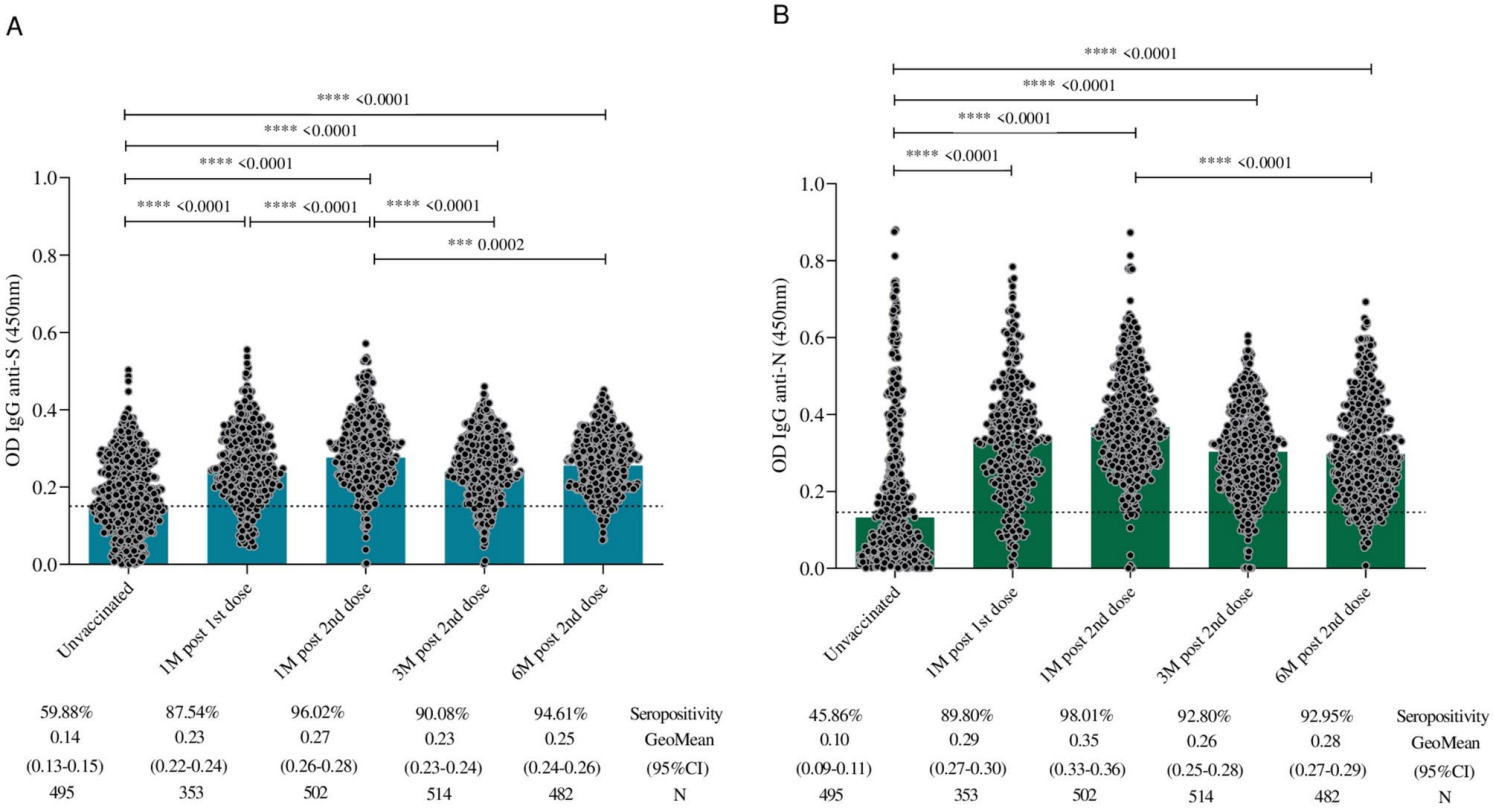
Kinetics of SARS-CoV-2 anti-S IgG (**A**) and anti-N IgG (**B**) levels at pre-vaccination, 1 month after the first dose, and 1, 3, and 6 months after receiving the second dose of CoronaVac. The cutoff of 0.1508 in (**A**) and 0.1460 in (**B**) is represented by dashed lines. The black dots represent individual data points of optical density (450 nm) for each vaccinated participant. The geometric mean titers (GMT) of IgG antibodies against S and N proteins is represented by blue and green bars, respectively. Statistical differences defined by Kruskal–Wallis and Mann–Whitney methods are represented for comparisons over time.

When evaluating the kinetics of total IgG antibodies against S and N proteins of SARS-CoV-2 (Fig. 2), a significant difference in anti-S titers (p < 0.0001) and anti-N IgG antibodies (p < 0.0001) was observed one month after completing the primary immunization. This difference persisted throughout the analyzed period of up to six months for both specific antibodies. Seropositivity rates were 96.0%, 90.0%, and 94.6%, respectively, at 1, 3, and 6 months for antibodies against the S protein of the virus. A similar profile was identified for the nucleocapsid protein, with seropositivity rates of 98.0%, 92.8%, and 92.9%, respectively, at 1, 3, and 6 months after receiving both doses of CoronaVac.

**Fig. 3:**
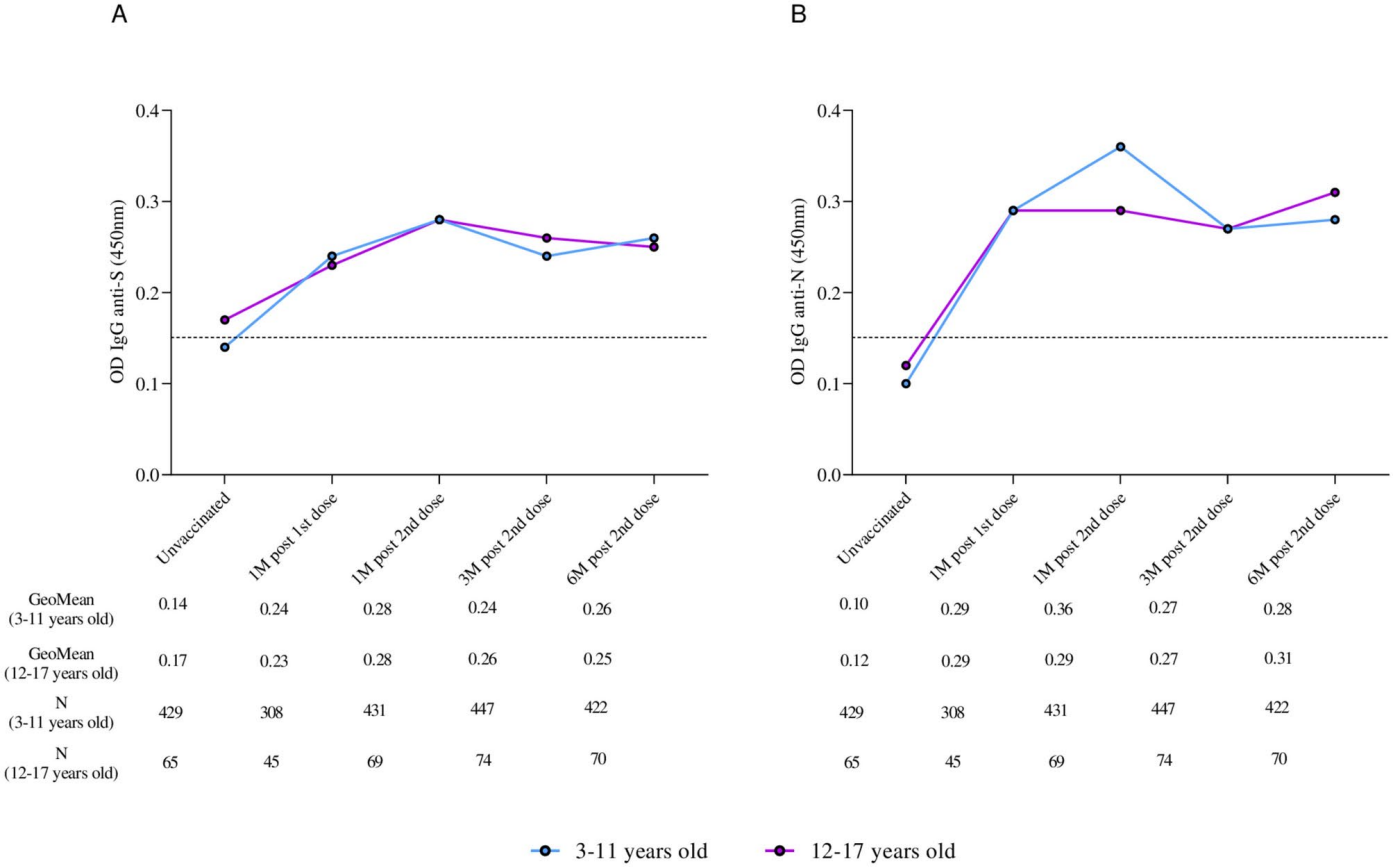
Kinetics of total IgG anti-S (**A**) and anti-N (**B**) antibody levels to SARS-CoV-2 at pre-vaccination, 1 month after the first dose, and 1, 3, and 6 months after receiving the second dose of CoronaVac separately for age groups (3–11 and 12–17 years old). The detection limit of 0.1508 in (**A**) and 0.1460 in (**B**) is represented by dashed lines. The colored dots represent the geometric mean of optical density (450 nm) for each vaccinated age group.

In the age group analysis (3–11 and 12–17 years old), the kinetics of total IgG antibodies to S and N proteins had the same response profile, with a significant increase in total antibodies one month after the second dose compared to pre-vaccination levels (Fig. 3).

**Fig. 4:**
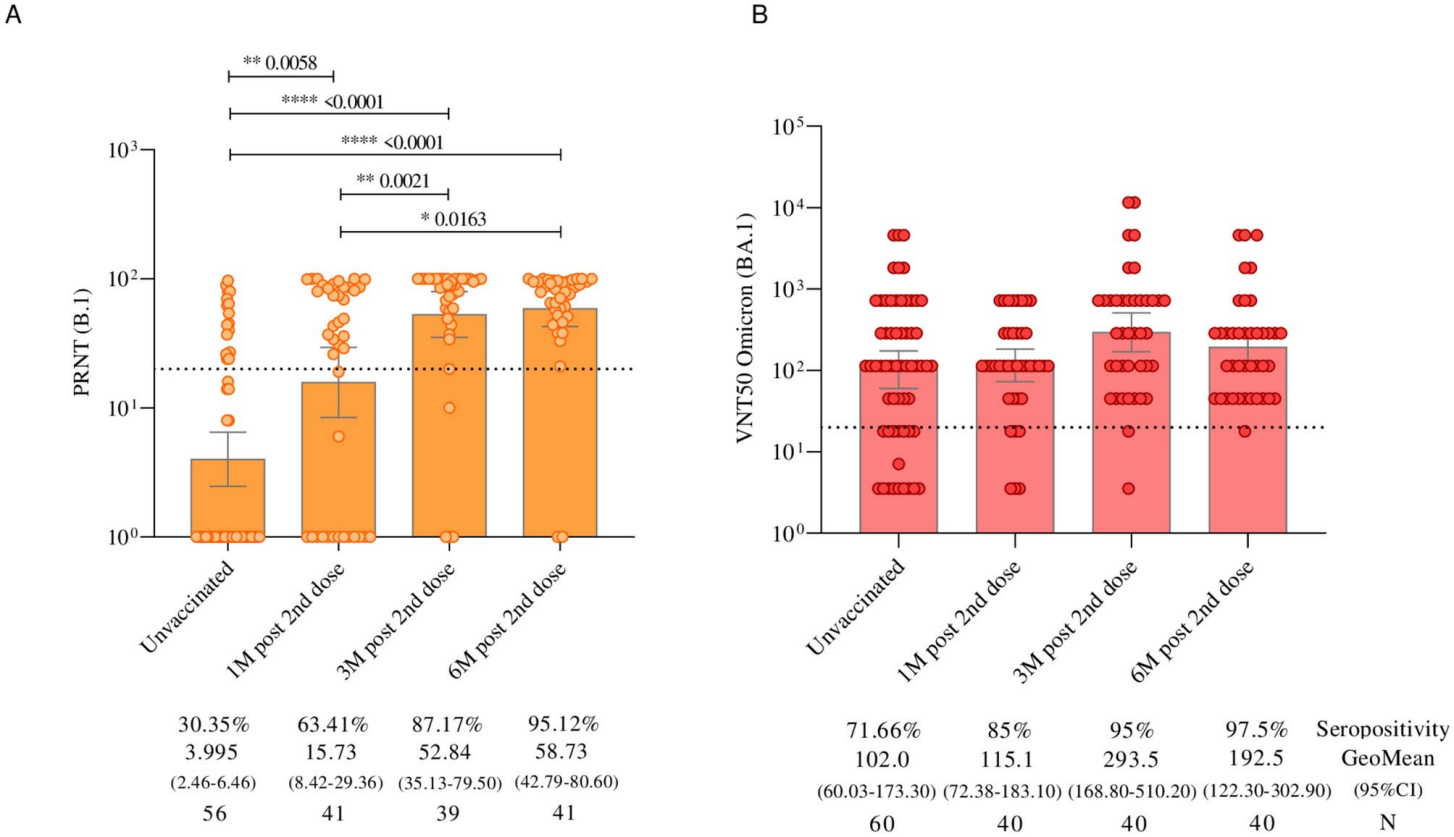
(**A**) Neutralizing antibodies detected by PRNT against the B.1 lineage of SARS-CoV-2 in children and adolescents vaccinated with CoronaVac. (**B**) Neutralizing antibodies detected by VNT50 against the Omicron variant (BA.1) of SARS-CoV-2 in children and adolescents vaccinated with CoronaVac. The cutoff for seropositivity, defined as 20, is represented by dashed lines. The geometric mean antibody titer is represented by bars. The colored points represent the individual results of each participant at different follow-up times in the study. Statistical differences, defined by Mann–Whitney tests, are presented for comparisons over time.

The levels of neutralizing antibodies determined by the PRNT assay using the B.1 lineage of SARS-CoV-2 (Fig. 4A) demonstrated a significant increase in seropositivity: from 30.3% at 1 month to 63.4%, 87.2% at 3 months, and 95.2% at 6 months (p < 0.0001) after the administration of the primary protocol. The mean assessment showed increases of 108.9% at 1 month, 187.9% at 3 months, and 213.9% at 6 months after vaccination, compared to the initial time point of this study.

Regarding the titers of neutralizing antibodies against Omicron variant (BA.1) determined by VNT50 (Fig. 4B), the seropositivity increased from 71.7% to 85% at 1 month, with a significant increase to 95% at 3 months (p = 0.0207), and 97.5% at 6 months after the second dose. The mean assessment showed increases of 18.6% at 1 month, 32.5% at 3 months, and 36.0% at 6 months after vaccination, compared to the first follow-up time.

**Fig. 5:**
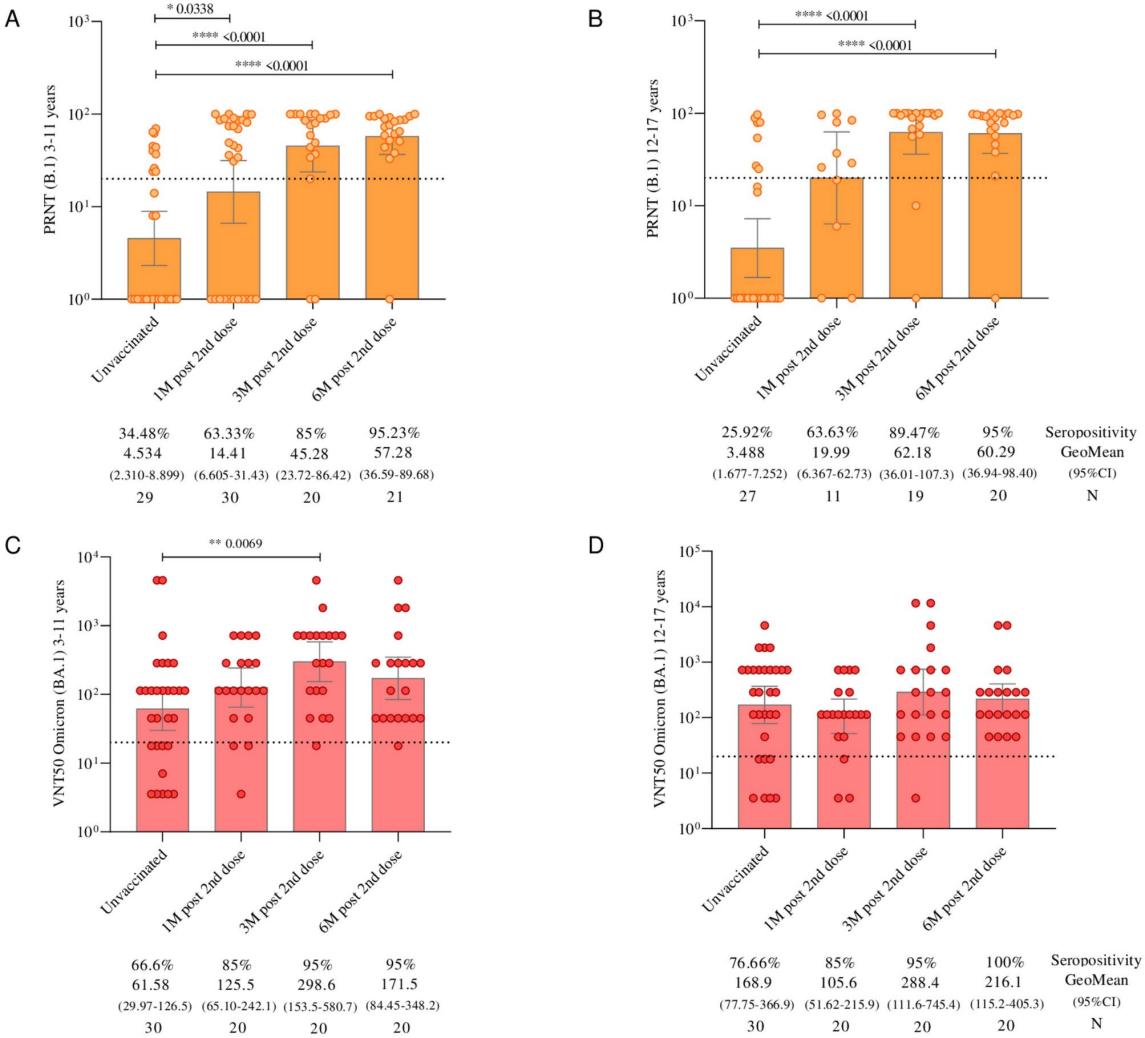
(**A**) Neutralizing antibodies to B.1 lineage of SARS-CoV-2 (detected by PRNT) in children aged 3 to 11 years vaccinated with CoronaVac. (**B**) Neutralizing antibodies detected by PRNT to B.1 lineage of SARS-CoV-2 in adolescents aged 12 to 17 years vaccinated with CoronaVac. (**C**) Neutralizing antibodies to Omicron variant (BA.1) (detected by VNT50) in children aging from 3 to 11 years vaccinated with CoronaVac. (**D**) Neutralizing antibodies to Omicron variant (BA.1) (detected by VNT50) in adolescents aging from 12 to 17 years vaccinated with CoronaVac. The cutoff for seropositivity, defined as 20, is represented by dashed lines. The geometric mean antibody titer is represented by bars. The colored points represent the individual results of each participant at different follow-up times in the study. Statistical differences, defined by Mann–Whitney tests, are presented for comparisons over time.

When analyzing neutralizing antibodies by age subgroups, both children (3–11 years) (Fig. 5A) and adolescents (12–17 years) (Fig. 5B) showed a significant increase in the detection of neutralizing antibodies against the B.1 lineage of SARS-CoV-2 at 3 and 6 months after the second dose, compared to pre-vaccination levels. However, neutralizing antibodies against the Omicron variant (BA.1) increased significantly only in children 3 months after the second dose of the CoronaVac (Fig. 5C and D), compared to pre-vaccination levels (p = 0.0069).

**Fig. 6:**
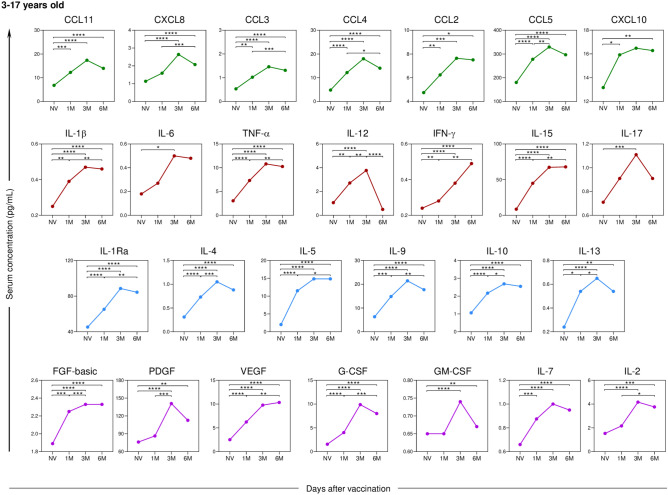
Kinetics of serum soluble mediators at one month (1 M), three months (3 M), and six months (6 M) after receiving two doses of CoronaVac in children and adolescents (3–17 years old) compared to the pre-vaccination period (Not Vaccinated—NV). The biomarkers were individually presented for chemokines (CXCL8, CCL11, CCL3, CCL4, CCL2, CCL5, and CXCL10); pro-inflammatory cytokines (IL-1β, IL-6, TNF-α, IL-12, IFN-γ, IL-15, and IL-17); regulatory cytokines (IL-1Ra, IL-4, IL-5, IL-9, IL-10, and IL-13); and growth factors (FGF-basic, PDGF, VEGF, G-CSF, GM-CSF, IL-7, and IL-2). Statistical differences by Mann–Whitney and ANOVA with significance levels of p < 0.05 are denoted by (*) between groups.

Serum soluble mediators from 173 participants who had no previous diagnosis of COVID-19 at pre-vaccination or during the 6-month follow-up revealed an increase in the levels of chemokines (CCL11, CCL3, CCL4, CCL2, CCL5, and CCL10), pro-inflammatory cytokines (IL-1β, TNF-α, IL-12, IFN-γ, and IL-15), regulatory cytokines (IL-1Ra, IL-4, IL-5, IL-9, IL-10, and IL-13), and growth factors (FGF-basic, PDGF, VEGF, G-CSF, IL-7, and IL-2) shortly after the first month compared to the pre-vaccination time point (Fig. 6). Furthermore, most of the biomarkers analyzed showed a significant increase between 1 and 6 months, except for the pro-inflammatory cytokine IL-12, which exhibited a decrease between the third and sixth months of the study follow-up.

**Fig. 7:**
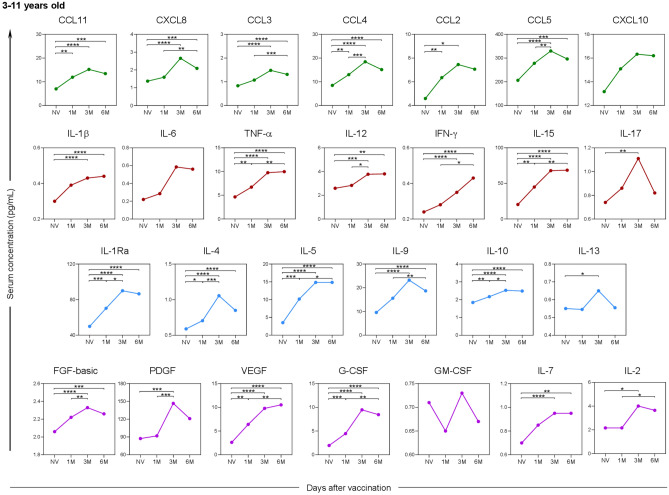
Kinetics of serum soluble mediators at one month (1 M), three months (3 M), and six months (6 M) after receiving two doses of CoronaVac in children (3–11 years old) compared to the pre-vaccination period (Not Vaccinated—NV). The biomarkers were individually presented for chemokines (CXCL8, CCL11, CCL3, CCL4, CCL2, CCL5, and CXCL10); pro-inflammatory cytokines (IL-1β, IL-6, TNF-α, IL-12, IFN-γ, IL-15, IL-17); regulatory cytokines (IL-1Ra, IL-4, IL-5, IL-9, IL-10, and IL-13); and growth factors (FGF-basic, PDGF, VEGF, G-CSF, GM-CSF, IL-7, and IL-2). Statistical differences by Mann–Whitney and ANOVA with significance levels of p < 0.05 are denoted by (*) between groups.

When analyzing the soluble serum biomarkers separately by age group, a similar profile was observed among children (Fig. 7) compared to the overall assessment of study participants, except for the biomarkers CXCL10 and IL-6, which did not show a statistically significant difference during the evaluated follow-up period.

**Fig. 8:**
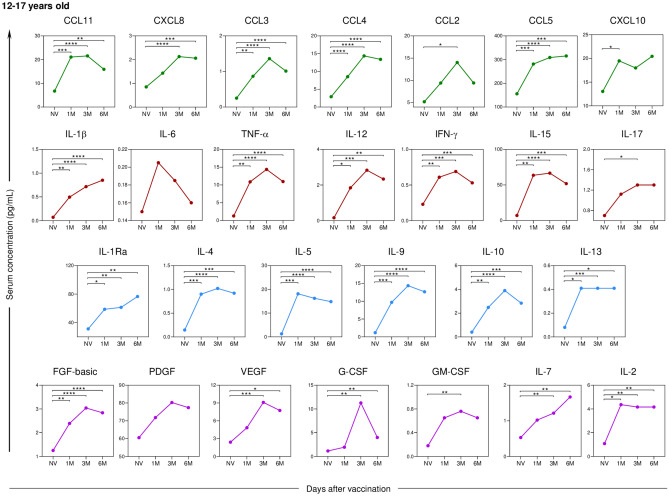
Kinetics of serum soluble mediators at one month (1 M), three months (3 M), and six months (6 M) after receiving two doses of CoronaVac in adolescents (12–17 years old) compared to the pre-vaccination period (Not Vaccinated—NV). The biomarkers were individually presented for chemokines (CXCL8, CCL11, CCL3, CCL4, CCL2, CCL5, and CXCL10); pro-inflammatory cytokines (IL-1β, IL-6, TNF-α, IL-12, IFN-γ, IL-15, IL-17); regulatory cytokines (IL-1Ra, IL-4, IL-5, IL-9, IL-10, and IL-13); and growth factors (FGF-basic, PDGF, VEGF, G-CSF, GM-CSF, IL-7, and IL-2). Statistical differences by Mann–Whitney and ANOVA with significance levels of p < 0.05 are denoted by (*) between groups.

As for adolescents (Fig. 8), besides the inflammatory cytokine IL-6, there was no statistically significant difference in the growth factor PDGF between any of the post-vaccination time points.

**Fig. 9:**
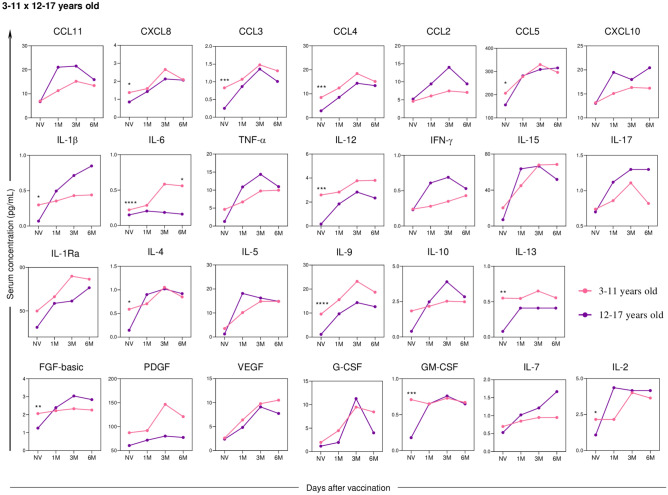
Differences in the kinetics of serum soluble mediators between children (3–11 years old) and adolescents (12–17 years old) at one month (1 M), three months (3 M), and six months (6 M) after receiving two doses of CoronaVac compared to the pre-vaccination period (Not Vaccinated—NV) are presented. The biomarkers were individually analyzed for chemokines (CXCL8, CCL11, CCL3, CCL4, CCL2, CCL5, and CXCL10); pro-inflammatory cytokines (IL-1β, IL-6, TNF-α, IL-12, IFN-γ, IL-15, IL-17); regulatory cytokines (IL-1Ra, IL-4, IL-5, IL-9, IL-10, and IL-13); and growth factors (FGF-basic, PDGF, VEGF, G-CSF, GM-CSF, IL-7, and IL-2). Statistical differences between groups were assessed using Mann–Whitney and ANOVA tests, with significance levels of p < 0.05 denoted by (*).

Comparing the cellular response between the age groups of 3–11 years and 12–17 years (Fig. 9), significantly higher levels of serum soluble mediators can be observed in children compared to adolescents, especially in the initial follow-up period of the study, except for the cytokine IL-6, which showed a statistically significant difference between these ages’ groups six months after the administration of the second dose.

### Safety Response: Identified AEs and SAEs

One hundred and ninety-two participants (30%) experienced adverse events (AEs), totaling three hundred seventy-nine (379) events, of which three occurring in infants (Table 2). Among these, 39.3% (149) were described as solicited systemic adverse events, 33% (125) were unsolicited adverse events, and 26.6% (101) were solicited local adverse events.

**Table 2:** Adverse events reported after the administration of CoronaVac in children and adolescents during a six-month follow-up.

	N	%
Adverse event reports		
Total number of participants with reports	192	30.0
Total adverse events reported	379	
Type of adverse event		
Solicited systemic adverse event	149	39.3
Unsolicited adverse event	125	33.0
Requested local adverse event	101	26.6
Not classified	4	1.1
Main adverse events reported		
Pain at the vaccine administration site	91	24.0
COVID-19	47	12.4
Fever	33	8.7
Cough	24	6.3
Runny nose	16	4.2
Cold	16	4.2
Symptoms of flu syndrome	15	4.0
Vomiting	10	2.6
Sore throat	6	1.6
Diarrhea	6	1.6
Sinusitis	5	1.3
Acute tonsillitis	4	1.1
Otitis	4	1.1
Odynophagia	3	0.8
Abdominal pain	2	0.5
Edema at the vaccine administration site	2	0.5
Malaise	2	0.5
Nausea	2	0.5
Allergy	2	0.5
Classification of the intensity of the adverse event		
1	265	69.9
2	88	23.2
3	11	2.9
4	8	2.1
Not classified	7	1.8
Causal relationship of the adverse event with the vaccine		
Not related	110	29.0
Certain	100	26.4
Unlikely	91	24.0
Possible	50	13.2
Probable	24	6.3
Not classified	4	1.1
Need for medical attention		
No	287	75.7
Yes	89	23.5
Not described	3	0.8

Among the major adverse events reported by study participants, 24% (91) experienced pain at the vaccine administration site, 8.7% (33) fever, and 4.2% (16) runny nose, with the majority of these being of intensity grade 1 (69.9%, 265).

Regarding the causal relationship of adverse events with the vaccine, 29% (110) were not related, 26.4% (100) were certain, 24% (91) were unlikely, 13.2% (50) were possible, and 6.3% (24) were probable, with most individuals not requiring medical and/or hospital care (75.7%, 287).

The recorded adverse events were mild and transient, except for seven Serious Adverse Events (SAEs) (Table 3), which were monitored by the study’s clinical team until resolution. Among the SAEs, three were unlikely to be related to the vaccine, and four were classified as not related. All participants had a favorable recovery outcome.

**Table 3:** Serious adverse events reported after the administration of the CoronaVac in children and adolescents during a six-month follow-up.

Local	Serious adverse event	Causality	Predictability	Severity Criterion	Clinical outcome
Serrana, SP	Acute Gastroenteritis	Unlikely	Not expected	Hospitalization	Recovered
Serrana, SP	Pre and post septal cellulite on the right	Unlikely	Not expected	Hospitalization	Recovered
Serrana, SP	Arm fracture	Not related	Not expected	Hospitalization	Recovered
Serrana, SP	Cellulitis	Not related	Not expected	Hospitalization	Recovered
Serrana, SP	Arm fracture	Not related	Not expected	Hospitalization	Not recovered
Belo Horizonte, MG	Asthmatic bronchiolitis	Unlikely	Not expected	Hospitalization	Recovered
Belo Horizonte, MG	Mastoiditis	Not related	Not expected	Hospitalization	Recovered

### Identification of SARS-CoV-2 by NGS

Active surveillance revealed two hundred and nine (209; 32.66%) suspected cases of SARS-CoV-2 infection, out of which fifty-six (56; 8.75%) were confirmed through RT-qPCR. There were no reports of hospitalizations or deaths. Eleven samples were submitted for sequencing (due to the Ct limit), all of which identified the Omicron variant, with 3 (27.27%) classified as Omicron (BA.2-like), 1 (9.09%) as Omicron (BA.5-like), and 7 (63.64%) as Omicron (Unassigned) (Tables 4 and 5).

**Table 4:** SARS-CoV-2-related infections, hospitalizations, and deaths among study participants.

Outcome	N	%
Monitoring children and adolescents	640	100.0
Suspected SARS-CoV-2 infection	209	32.7
Confirmed COVID-19 diagnosis	56	8.75
Hospitalizations	0	0.00
Deaths	0	0.00

**Table 5:** Identification of SARS-CoV-2 variant by NGS among study participants.

Identification of SARS-CoV-2 by NGS	N	%
NGS performed	11	100
Omicron (BA.2-like)	3	27,27
Omicron (BA.5-like)	1	9,09
Omicron (Unassigned)	7	63,64

## Discussion

Data pertaining to immunogenicity, as assessed through total antibody levels by anti-SARS-CoV-2 ELISA, revealed that most children and adolescents exhibited seropositivity for anti-S IgG prior to vaccination, while 46% displayed seropositivity for anti-N IgG, indicating prior exposure to SARS-CoV-2 before immunization.

Epidemiological data published during the period of this study demonstrated peaks of COVID-19 transmission in Brazil, particularly in the southeastern region of the country, where there was a prevalence of 58.4% of SARS-CoV-2 infections among severe acute respiratory syndrome (SARS) cases, according to data published at InfoGripe^23^. Furthermore, a study assessing the circulation of SARS-CoV-2 in children in Brazil from April 2020 to July 2022 reported a higher risk of infection in children from symptomatic family adults, usually the mother, reinforcing the importance of vaccination across all age groups^24^.

A comprehensive statistical analysis of the collective data indicated a significant rise in titers of anti-S IgG antibodies and anti-N IgG antibodies, as well as in seropositivity rates, one month after completing the primary immunization course with two doses of CoronaVac. Similar findings were reported by Fernandes et al.^25^, wherein all children in the study exhibited a substantial increase in antibody titers induced one month after vaccination, underscoring a robust serological response to a single dose in a pediatric population up to five years old, with no reports of severe adverse effects.

Despite a reduction in the overall mean antibody titers observed at three months post-vaccination in our study, these averages remained consistently high (above the detection limit of the reference assay) throughout the study period, demonstrating the durability and persistence of the total antibody response. The seropositivity rate reached 90% for anti-S IgG in the third month and 95% after six months of follow-up. Similarly, seropositivity rates reached 93% and 99% for anti-N IgG in the third- and sixth-months post-vaccination. Evaluation of the geometric mean titers of anti-S and anti-N IgG antibodies among participants grouped by age revealed a consistent pattern of seropositivity, confirming a substantial increase in total antibodies against SARS-CoV-2 within the first month after vaccination, followed by a stable immune response over the course of the six-month study.

These outcomes align with those reported by Rosa et al.^26^, indicating that 97% of adolescents aged 11 to 17 displayed anti-S IgG antibodies against SARS-CoV-2 after receiving two doses of CoronaVac. Moreover, the same adolescents were assessed for anti-N IgG as secondary immunogenicity markers, revealing a high seropositivity rate of 98%. Additionally, in accordance with findings from the Immunita-02 study, previously published data indicated that over 96% of children and adolescents aged 3 to 17 years exhibited specific antibodies to SARS-CoV-2 after 28 days of receiving two doses of CoronaVac^27^.

Virus neutralization assays showed that a significant percentage of children and adolescents displayed seropositivity to the B.1 lineage prior to vaccination. This percentage increased after the first dose and continued to rise following the second dose. Regarding neutralizing antibodies to the Omicron variant, most of the children and adolescents being monitored showed seropositivity before their first dose, indicating previous infection. After vaccination, they maintained significant seropositivity over time, indicating a sustained neutralizing response to the Omicron variant across different age groups. Good neutralizing antibody responses were also noted following the administration of CoronaVac in previously published phase 1 and 2 studies, which reported the vaccine’s good tolerability and safety, along with satisfactory humoral responses in children and adolescents aged 3 to 17 years. Additionally, it was observed that the neutralizing antibody titers induced by the 3.0 μg dose exceeded those of the 1.5 μg dose. These findings support the use of the 3.0 μg dose in a two-dose immunization regimen for further investigations in the same age group^27^.

We chose to use individuals without a history of prior infection for the VNT assays. However, our data show that a significant portion of them already had neutralizing antibodies before vaccination, both for the Omicron variant and the ancestral B lineage, indicating previous, likely asymptomatic exposure. A meta-analysis study published in the Journal of Medical Virology compiled data on asymptomatic infections, revealing that during waves of Omicron variant infections, children and adolescents had a high proportion of asymptomatic cases (82%), while in the elderly (already widely vaccinated at that time), this proportion was lower (62%)^28^. These data reinforce the importance of vaccination among children and adolescents, as they may contribute to the ongoing circulation of SARS-CoV-2^28^.

Regarding the cellular response data demonstrated in this study, in general, a similar profile was observed among the age groups, with occasional differences in pro-inflammatory cytokines when comparing the profiles of children to adolescents from the third to the sixth month of follow-up. An initial inflammation observed triggered by vaccination is a premise for the development of a robust cellular response. It is noticeable that there was an increasing release of soluble pro-inflammatory and regulatory mediators in the first three months post-vaccination, with the maintenance of the response at six months of post-vaccination follow-up. Hence, it is evident that a robust and enduring cellular immune response, characterized by a combination of pro-inflammatory and regulatory elements, persists for up to six months after the administration of two doses of the CoronaVac in the children and adolescents observed within the scope of this study.

Promising results regarding the cellular immune response in children and adolescents who received CoronaVac have also been reported, as described by Soto and collaborators^28^. Their findings highlighted a significant increase in CD4 + AIM + T-cells in response to the structural proteins of the virus. In the 12 to 17-year-old group, there was a notable expansion in the activation of CD4 + T-cells in response to all the structural proteins of SARS-CoV-2, indicating that CoronaVac, as an inactivated virus vaccine, stimulates cellular immunity not only against the Spike (S) protein but also against the membrane (M) and nucleocapsid (N) proteins. Furthermore, an increase in IL-2 secretion in response to the S and N proteins was observed in both age groups, with this increase being particularly pronounced in individuals aged 12 to 17 years in relation to the M protein^29^. The vaccine also promoted a pro-inflammatory profile, with an increase in IFN-γ and no increase in IL-4, in both the 3 to 11-year-old group and the 12 to 17-year-old group. Additionally, an increase in the frequency of memory CD4 + AIM + T-cells in response to the SARS-CoV-2 proteins was noted, with a slight intensification observed in individuals aged 12 to 17 years, suggesting that the vaccine may induce long-lasting CD4 + T-cell responses^29^.

Among the 640 children and adolescents monitored, one third presented suspected cases of COVID-19. After conducting RT-qPCR tests, 56 cases of SARS-CoV-2 infections were confirmed, with no instances of moderate or severe COVID-19 necessitating hospitalization among the pediatric population. Of the swab samples collected from COVID-19 positive participants, 11 met the criteria for NGS sequencing, all of which were identified as belonging to the Omicron variant sub-lineages, including BA.2 and BA.5.

After vaccination, mostly mild and transient adverse effects were reported, with pain at the injection site being the most common issue. The few serious adverse events observed were unrelated to the vaccine and included conditions like acute gastroenteritis, cellulitis, fractures, and asthmatic bronchiolitis. Similar outcomes were observed in phase 1 and 2 studies described by Han and collaborators^27^, where most post-vaccination adverse reactions were mild and transient, with pain at the injection site being the most frequently reported event (73.1% of 550 participants). Furthermore, in a phase 3 study conducted in Chile, the primary adverse event reported after the first and second doses was also pain at the injection site. This study also concluded that CoronaVac was safe and immunogenic in individuals aged 3 to 17 years, inducing the production of neutralizing antibodies and activation of CD4 + T-cells to SARS-CoV-2 variants^29^.

Recently published data on the effectiveness of the CoronaVac against COVID-19 in Brazilian children (aged 6 to 11 years), during a period of high Omicron variant (B.1.1.529 lineage) circulation (January 21, 2022, to April 15, 2022), indicated an estimated vaccine effectiveness of 39.8% (95% CI 33.7–45.4) against symptomatic infection at ≥ 14 days post-second dose^30^. For hospitalization, vaccine effectiveness was 59.2% (95% CI 11.3–84.5) at ≥ 14 days^30^. Conversely, noteworthy results emerged from the evaluation of CoronaVac effectiveness in Chile, involving a large national prospective cohort of approximately two million children and adolescents aged 6 to 16 years. The estimated effectiveness stood at 74.5% (95% CI, 73.8–75.2), 91.0% (95% CI, 87.8–93.4), and 93.8% (95% CI, 87.8–93.4) for the prevention of COVID-19, hospitalization, and ICU admission, respectively^31^. For children aged 6 to 11 years, vaccine effectiveness was 75.8% (95% CI, 74.7–76.8) against COVID-19 and 77.9% (95% CI, 61.5–87.3) against hospitalization^31^.

Another study assessing CoronaVac effectiveness in children aged 3 to 5 years, conducted during the Omicron (B.1.1.529 lineage) outbreak in Chile, revealed estimated effectiveness of 38.2% (95% CI, 36.5–39.9) against symptomatic COVID-19, 64.6% (95% CI, 49.6–75.2) against hospitalization, and 69.0% (95% CI, 18.6–88.2) against ICU admission^31^. The study concluded that despite the modest effectiveness against symptomatic COVID-19, CoronaVac vaccination provided robust protection against severe COVID-19 in this age group^32^.

This study adopted a prospective observational design, which provided valuable insights into the immune response and effectiveness of CoronaVac in children and adolescents. However, it is important to note that the study results are based on a sample recruited from two locations in southeastern Brazil. Therefore, it is important to consider that sociodemographic and health characteristics of these regions may differ significantly from other parts of the country. While the study included a valuable cohort, a larger sample size would provide greater statistical power and more robust conclusions. Although this study followed participants for a period of six months after the second dose of CoronaVac, the long-term impact of vaccination should be assessed. Hence, further studies are needed to evaluate the persistence of immune response and safety over longer durations in this age group. Additionally, to complement the immunogenicity data of this study, it is recommended to conduct broader assessments such as specific T and B cell responses to SARS-CoV-2, to obtain more comprehensive insights into the immune response following vaccination.

## Conclusion

In conclusion, the data evaluation from this study underscores a robust immunogenic response elicited by CoronaVac and its sustained nature in children and adolescents aged 3 to 17 years over a period of up to six months following the completion of the primary vaccination regimen. The vaccine notably and comprehensively stimulated the production of specific IgG antibodies targeting the S and N proteins of the virus, as well as the generation of neutralizing antibodies effective against both the B.1 lineage and the Omicron variant (BA.1). Furthermore, this vaccination regimen triggered a substantial mixed-pattern cellular response with widespread release of pro-inflammatory and regulatory mediators, highlighting the vaccine’s safety and efficacy in mitigating COVID-19 in children and adolescents.

## Supplementary Information

Supplementary Information 1.

Supplementary Information 2.

Supplementary Information 3.

Supplementary Information 4.

## Data Availability

The datasets used and/or analyzed during the current study available from the corresponding author on reasonable request. Raw reads are available in ENA under the project accession PRJEB85438.
