# Supplementary material for: Immunogenicity and safety of CoronaVac vaccine in children and adolescents (Immunita-002, Brazil): A phase IV six-month follow up
Source: Sci Rep. 2025 Jul 2;15:23040. doi: 10.1038/s41598-025-94596-9 (PMC12215048; doi:10.1038/s41598-025-94596-9)
Supplement: Supplementary file 1 — Supplementary Information 1. [file 41598_2025_94596_MOESM1_ESM.docx]

**Supplementary table 1.** Classification of intensity for requested local clinical adverse events.

| **Requested local adverse event** | **Grade 1** | **Grade 2** | **Grade 3** | **Grade 4** |  |
| --- | --- | --- | --- | --- | --- |
| Pain at the site of administration of the investigational product | Does not interfere with daily activities | Repeated use of non-narcotic analgesic >24 hours OR | Any use of narcotic analgesic OR | Emergency room visit* OR |  |
|  |  | Slightly interferes with daily activities | Impairs daily activities | Hospitalization |  |
| Erythema at the site of administration of the investigational product ^†^ | 25 – 50 mm | 51 – 100 mm | > 100 mm | Necrosis OR |  |
|  |  |  |  | Exfoliative dermatitis |  |
| Swelling at the site of administration of the investigational product | 25 – 50 mm | 51 – 100 mm OR | > 100 mm OR | Necrosis |  |
|  |  | Interferes with daily activities | Impairs daily activities |  |  |
|  |  |  |  |  |  |
| Induration at the site of administration of the investigational product | 25 – 50 mm | 51 – 100 mm OR | > 100 mm OR | Necrosis |  |
|  |  | Interferes with daily activities | Impairs daily activities |  |  |
| Pruritus at the site of administration of the investigational product | Does not interfere with daily activities | Interferes with daily activities | Impairs daily activities | Emergency room visit* OR |  |
|  |  |  |  | Hospitalization |  |

* Need for 12 hours or more of hospitalization in the ward or emergency room for the management of the adverse event

† The recorded value was measured at the site of the largest diameter and as a continuous variable.
