# Supplementary material for: Immunogenicity and safety of CoronaVac vaccine in children and adolescents (Immunita-002, Brazil): A phase IV six-month follow up
Source: Sci Rep. 2025 Jul 2;15:23040. doi: 10.1038/s41598-025-94596-9 (PMC12215048; doi:10.1038/s41598-025-94596-9)
Supplement: Supplementary file 2 — Supplementary Information 2. [file 41598_2025_94596_MOESM2_ESM.docx]

**Supplementary table 2.** Classification of intensity for requested systemic clinical adverse events and signs and symptoms in case of fever and suspected COVID-19.

| **Requested systemic adverse event** | **Grade 1** | **Grade 2** | **Grade 3** | **Grade 4** |
| --- | --- | --- | --- | --- |
| Fever | 37.8°C – 38.4°C | 38.5°C – 38.9°C | 39.0°C – 40.0°C | >40°C |
| Nausea | Does not interfere with daily activities OR | Slightly interferes with daily activities OR | Impairs daily activities, requires intravenous hydration | Emergency room visit* OR |
|  | 1 to 2 episodes in 24 hours | More than 2 episodes in 24 hours |  | Hospitalization OR |
|  |  |  |  | Hypovolemic shock |
| Vomiting | Does not interfere with daily activities OR | Slightly interferes with daily activities OR | Impairs daily activities, requires intravenous hydration | Emergency room visit* OR |
|  | 1 to 2 episodes in 24 hours | More than 2 episodes in 24 hours |  | Hospitalization OR |
|  |  |  |  | Hypovolemic shock |
| Diarrhea | 2 - 3 loose stools in 24 hours | 4 - 5 stools in 24 hours | 6 or more liquid stools or requires IV hydration in urgent care | Emergency room visit* OR |
|  |  |  |  | Hospitalization |
| Headache | Does not interfere with daily activities | Repeated use of non-narcotic analgesic >24 hours OR | Any use of narcotic analgesic OR | Emergency room visit* OR |
|  |  | Slightly interferes with daily activities | Impairs daily activities | Hospitalization |
| Fatigue | Does not interfere with daily activities | Slightly interferes with daily activities | Impairs daily activities | Emergency room visit* OR |
|  |  |  |  | Hospitalization |
| Myalgia | Does not interfere with daily activities | Slightly interferes with daily activities | Impairs daily activities | Emergency room visit* OR |
|  |  |  |  | Hospitalization |
| Chills | Mild sensation of cold; shivering, teeth chattering | Moderate sensation of whole-body shaking, requires opioid use | Severe or prolonged, unresponsive to opioids | ----- |
| Anorexia | Loss of appetite without changes in eating habits | Altered oral intake without significant weight loss or malnutrition; oral nutritional supplements recommended | Associated with significant weight loss or malnutrition (inadequate intake of calories and/or oral fluids); tube feeding, or complete parenteral nutrition indicated | ----- |
| Cough | Mild symptoms: intervention without prescription indicated | Moderate symptoms, medical intervention indicated; limits instrumental activities of daily living | Severe symptoms; limits activities of daily living self-care | ----- |
| Arthralgia | Mild pain | Moderate pain; limits instrumental activities of daily living | Severe pain; limits activities of daily living self-care | ----- |
| Pruritus | Mild or localized. May require topical medication | Intense or widespread; intermittent. Skin changes due to scratching (edema, papules, excoriations, lichenification, crusts). Interferes with daily activities. May require oral medication. | Intense or widespread; intermittent. Impairs self-care, daily activities, or sleep. May require systemic corticosteroid or immunosuppressant. | ----- |
| Skin rash exanthema^†^ | Present but asymptomatic | Symptomatic (itching/pain) but interferes little with daily activities | Symptomatic, impairs daily activities | Emergency room visit* OR |
|  |  |  |  | Hospitalization |
| Allergic reaction | No systemic intervention indicated | Oral intervention indicated | Bronchospasm: hospitalization indicated for clinical sequelae; intravenous intervention indicated | Life-threatening consequences: urgent intervention indicated |

* Need for 12 hours or more of hospitalization in the ward or emergency room for management of the adverse event.

^†^ Specificity about the rash being localized in a specific body region or if it is generalized.
