# Supplementary material for: Immunogenicity and safety of CoronaVac vaccine in children and adolescents (Immunita-002, Brazil): A phase IV six-month follow up
Source: Sci Rep. 2025 Jul 2;15:23040. doi: 10.1038/s41598-025-94596-9 (PMC12215048; doi:10.1038/s41598-025-94596-9)
Supplement: Supplementary file 3 — Supplementary Information 3. [file 41598_2025_94596_MOESM3_ESM.docx]

**Supplementary table 3.** Classification of the intensity of unsolicited clinical adverse events and other signs and symptoms in case of fever and suspicion of COVID-19.

| **Unsolicited adverse event** | **Grade 1** | **Grade 2** | **Grade 3** | **Grade 4** |
| --- | --- | --- | --- | --- |
| Respiratory rate | 17-20 breaths per minute | 21-25 breaths per minute | >25 breaths per minute | Intubation |
| Dyspnea | Shortness of breath with moderate exertion | Shortness of breath with minimal exertion; limiting instrumental activities of daily living | Shortness of breath at rest; limiting self-care activities of daily living | Life-threatening: Requires urgent intervention |
| Nasal congestion | Mild symptoms: no medical intervention needed | Moderate symptoms: medical intervention indicated | Associated with bloody nasal discharge or epistaxis | ----- |
| Anosmia | Present | ----- | ----- | ----- |
| Disgeusia / Ageusia | Altered taste, but no change in diet | Altered taste with changes in diet (oral supplements); unpleasant or harmful taste; loss of taste | ----- | ----- |
| Disease or Clinical Adverse Event (as defined by applicable standards) and other signs and symptoms | Does not interfere with daily activities | Interferes slightly with daily activities and does not require medical intervention | Impedes daily activities and requires medical intervention | Emergency room visit* OR |
|  |  |  |  | Hospitalization |

* Need for 12 hours or more of hospitalization in the ward or emergency room for the management of the adverse event

† The recorded value was measured at the site of the largest diameter and as a continuous variable.
