# Supplementary material for: Immunogenicity and safety of CoronaVac vaccine in children and adolescents (Immunita-002, Brazil): A phase IV six-month follow up
Source: Sci Rep. 2025 Jul 2;15:23040. doi: 10.1038/s41598-025-94596-9 (PMC12215048; doi:10.1038/s41598-025-94596-9)
Supplement: Supplementary file 4 — Supplementary Information 4. [file 41598_2025_94596_MOESM4_ESM.docx]

**Supplementary table 4.** Causal relationship classification of adverse events with the investigational product.

| **Reasonable causal relationship** | | | **Non-reasonable causal relationship** | |
| --- | --- | --- | --- | --- |
| **Adverse event considered as adverse reaction** | | | **The adverse event cannot be considered as adverse reaction** | |
| **Certain** | **Probable** | **Possible** | **Unlikely** | **Not related** |
| Event or abnormal laboratory test finding, with a plausible temporal relationship to the administration of the intervention | An adverse event, including an abnormal laboratory test finding, with a reasonable temporal relationship to the administration of the intervention | A clinical event, including an abnormal laboratory test finding, with a reasonable temporal relationship to the administration of the intervention | A clinical event, including an abnormal laboratory test finding, which due to the timing of the intervention administration, makes a relationship unlikely but not impossible | A clinical event, including an abnormal laboratory test finding, which due to the timing of the intervention administration, makes any relationship nonexistent |
| Cannot be explained by concomitant disease or other intervention or medication; | Unlikely to be attributed to a concomitant disease or other intervention or medication | It can also be explained by concomitant disease or other interventions or medications | Another disease or another medication provides a plausible explanation | Another disease or another medication provides a plausible explanation |
| The event is pharmacologically or phenomenologically defined (an objective and specific disorder or a pharmacologically recognized phenomenon) |  |  |  |  |
| The response to discontinuation or withdrawal is plausible (pharmacologically, pathologically) | The response to discontinuation or withdrawal is clinically reasonable | Lack of information or still unclear about treatment discontinuation or withdrawal | ----- | ----- |
| Satisfactory reexposure, if necessary | No reexposure required | ----- | ----- | ----- |

* Need for 12 hours or more of hospitalization in the ward or emergency room for the management of the adverse event

† The recorded value was measured at the site of the largest diameter and as a continuous variable.
